# Genomic Analysis of *Serratia plymuthica* MBSA-MJ1: A Plant Growth Promoting Rhizobacteria That Improves Water Stress Tolerance in Greenhouse Ornamentals

**DOI:** 10.3389/fmicb.2021.653556

**Published:** 2021-05-11

**Authors:** Nathan P. Nordstedt, Michelle L. Jones

**Affiliations:** Department of Horticulture and Crop Science, Ohio Agricultural Research and Development Center, The Ohio State University, Wooster, OH, United States

**Keywords:** drought, floriculture, horticulture, osmoprotectants, plant growth promoting rhizobacteria, plant-microbe interaction, vitamins, whole-genome sequence

## Abstract

Water stress decreases the health and quality of horticulture crops by inhibiting photosynthesis, transpiration, and nutrient uptake. Application of plant growth promoting rhizobacteria (PGPR) can increase the growth, stress tolerance, and overall quality of field and greenhouse grown crops subjected to water stress. Here, we evaluated *Serratia plymuthica* MBSA-MJ1 for its ability to increase plant growth and quality of *Petunia* × *hybrida* (petunia), *Impatiens walleriana* (impatiens), and *Viola* × *wittrockiana* (pansy) plants recovering from severe water stress. Plants were treated weekly with inoculum of MBSA-MJ1, and plant growth and quality were evaluated 2 weeks after recovery from water stress. Application of *S. plymuthica* MBSA-MJ1 increased the visual quality and shoot biomass of petunia and impatiens and increased the flower number of petunia after recovery from water stress. In addition, *in vitro* characterizations showed that MBSA-MJ1 is a motile bacterium with moderate levels of antibiotic resistance that can withstand osmotic stress. Further, comprehensive genomic analyses identified genes putatively involved in bacterial osmotic and oxidative stress responses and the synthesis of osmoprotectants and vitamins that could potentially be involved in increasing plant water stress tolerance. This work provides a better understanding of potential mechanisms involved in beneficial plant-microbe interactions under abiotic stress using a novel *S. plymuthica* strain as a model.

## Introduction

Abiotic stresses negatively impact a multitude of biochemical and physiological responses in crops, reducing plant growth, performance, and quality (Anjum et al., 2017; Nordstedt et al., 2020). Water stress, in particular, can permanently damage the photosynthetic health of plants, leading to a reduction in plant size and yield (Armitage et al., 1983; Zargar et al., 2017; Liang et al., 2020; Nordstedt and Jones, 2020). A decrease in available water leads to a reduction in total nutrient uptake and increases in cellular oxidative damage that inhibit plant growth (Farooq et al., 2009). Although the negative impact of water stress on the yield of food crops is well-documented, water stress can also decrease the quality of high-value ornamental crops grown in containers, which are more prone to rapid fluxes in water availability (Nordstedt et al., 2020). Water stress reduces the marketability of ornamental crops by negatively impacting plant size, flowering, and leaf color (Sánchez-Blanco et al., 2009). In addition to reducing the appearance of these high-value crops, damage to photosynthetic health can lead to reduced transplant success and plant vigor in the customer’s garden (Armitage et al., 1983; Sun et al., 2020).

Similar to plants, microbial communities are strongly influenced by water stress, which creates unfavorable osmotic conditions and increases competition for resources (Makhalanyane et al., 2015; Ngumbi and Kloepper, 2016). As water content in the rhizosphere decreases, bacteria must adapt by accumulating solutes, producing exopolysaccharides, and/or producing spores to survive. Fortunately, many of the strategies that rhizospheric bacteria utilize to cope with water stress can also be beneficial in helping their plant host overcome severe water stress. These beneficial bacteria are termed plant growth promoting rhizobacteria (PGPR), and they can increase plant growth and crop quality under a variety of environmental stresses including water stress (Ngumbi and Kloepper, 2016; Vurukonda et al., 2016; Backer et al., 2018).

These plant-microbe interactions are established *via* root exudates that are secreted into the rhizosphere (Lugtenberg and Kamilova, 2009). Root exudates comprise a variety of compounds that recruit and support microbial growth, such as sugars, amino acids, organic acids, secondary metabolites, carbon dioxide, and water. Plants secrete these exudates into the rhizosphere to recruit and support microbial growth, and in return PGPR can modulate phytohormone levels, influence root architecture, synthesize compounds such as osmoprotectants or vitamins, and increase the availability of nutrients to plants (Ngumbi and Kloepper, 2016). The composition of these root exudates changes during times of water stress, potentially optimizing conditions for PGPR to survive and continue supporting their plant host (Gargallo-Garriga et al., 2018).

In this work, we have identified *Serratia plymuthica* MBSA-MJ1 as a PGPR that improves plant growth and flowering of ornamental plant species after recovery from severe water stress. *Serratia* are a group of widespread gram-negative bacteria found in water, soil, and animals. The nature of *Serratia* species ranges from parasitic to mutualistic, however, many share the ability to survive under diverse environmental conditions and compete with other microorganisms to successfully colonize within their ecological niche (Petersen and Tisa, 2013). In addition to being found in a variety of environments, plant-associated *Serratia* spp. are also found to colonize a variety of plant hosts in the endosphere, phyllosphere, and rhizosphere (Benhamou et al., 2000; De Vleesschauwer and Höfte, 2007; Müller et al., 2013; Martínez et al., 2018).

Strains of *S. plymuthica* are most commonly associated with plant roots and have been isolated from a variety of plant hosts, including oilseed rape, poplar trees, wheat, potato, pumpkin, maize, and rice (Åström and Gerhardson, 1988; Gyaneshwar et al., 2001; Berg et al., 2002, 2006; van der Lelie et al., 2009; Garbeva et al., 2012; Müller et al., 2013; Neupane, 2013). Because of their association with plant roots, they have been studied extensively for their use as both biocontrol agents against soil-dwelling phytopathogens and as PGPR. Their efficacy as both a biocontrol agent and a PGPR is commonly attributed to the production of antimicrobial compounds, phytohormones, and secondary metabolites (Lavania et al., 2006; Matilla et al., 2016). Most reports of plant-associated *S. plymuthica* strains show evidence of biocontrol activity (Berg, 2000; Pang et al., 2009; Czajkowski and van der Wolfa, 2012; Garbeva et al., 2012; Müller et al., 2013; Adam et al., 2016), while some also report the additional ability to stimulate plant growth (Neupane, 2013; Li et al., 2020). Recently, *S. plymuthica* strains have been reported to colonize *Arabidopsis thaliana* (Proença et al., 2019) and stimulate the growth of water stressed *Ziziphus jujuba* (Zhang et al., 2020). The strain *Serratia* sp. 1–9 stimulates growth of wheat plants subjected to severe water stress (Wang et al., 2014), while other *Serratia* spp. stimulate plant growth under salt stress (El-Esawi et al., 2018), in heavy metal contaminated soils (Koo and Cho, 2009), and under low temperature stress (Zhang et al., 1997; Kang et al., 2015).

These beneficial plant-microbe interactions are very complex, and the complexity contributes to a lack of clarity as to how some bacteria increase plant growth and stress tolerance. These bacteria must be able to survive under the stressful environmental conditions that are impacting their plant host, while colonizing the rhizosphere or the plant tissue (i.e., endophytes) without causing disease symptoms. The microbial symbiont can then begin positively influencing plant growth and stress tolerance by modulating phytohormone levels, increasing nutrient availability, or producing osmoprotectants and vitamins for plant utilization. However, the mechanisms that are employed can range widely between different strains of PGPR. Whole-genome sequencing data provide insight into the dynamic mechanisms that PGPR might be utilizing in these complex interactions. To date there are over 1,000 *Serratia* spp. reference genomes publicly available, however, there are a limited number of references available for plant-associated strains. Additionally, there are currently no publicly available genome data for *S. plymuthica* strains that increase growth of water stressed plants. Therefore, the genomic analysis completed here, coupled with the *in vitro* and *in planta* experiments provide support for pathways or biochemical processes that *S. plymuthica* MBSA-MJ1 might be utilizing to survive, colonize, and ultimately increase plant growth under water stress. Identification of putative genes underlying the plant growth-promoting traits of this novel bacterium will lead to a better understanding of how bacteria can influence plant growth during water stress and contribute to future work in functional gene analysis.

## Materials and Methods

### Bacterial Strain

*Serratia plymuthica* MBSA-MJ1 is part of a bacteria collection from the laboratory of Dr. Christopher Taylor (The Ohio State University; Aly et al., 2007), although the exact source of this specific strain is unknown. MBSA-MJ1 was previously evaluated for its ability to reduce the severity of *Botrytis cinerea* infection in *Petunia* × *hybrida* (South et al., 2020).

### Water Stress Greenhouse Trial

The ability of *S. plymuthica* MBSA-MJ1 to increase the quality and growth of plants after recovery from water stress was evaluated using a previously established *in planta* greenhouse trialing protocol (Nordstedt et al., 2020). Greenhouse conditions were set to maintain canopy temperature at 24/18°C (day/night). Supplemental lighting was provided with metal halide and high-pressure sodium lights (GLX/GLS e-systems GROW lights, PARSource, Petaluma, CA, United States) to provide a 16 h photoperiod and maintain light levels above 250 mmol m^−2^ s^−1^. The experiment included three economically important ornamental crops including: *Petunia* × *hybrida* “Picobella Blue” (petunia; Syngenta Flowers Gilroy, CA), *Impatiens walleriana* “Super Elfin Ruby” (impatiens; PanAmerican Seed, West Chicago, IL), and *Viola* × *wittrockiana* “Delta Pure Red” (pansy; Syngenta Flowers). Seeds were sown for each of the plant species and transplanted after 3 weeks into 11.4 cm diameter pots containing Pro-Mix PGX potting media (Premier Tech Horticulture, Quakertown, PA, United States). Plants were arranged in a Randomized Complete Block Design (RCBD) with one plant per block with 13, 14, and 18 blocks for petunia, pansy, and impatiens, respectively. Plants were fertilized at each irrigation with a water-soluble fertilizer at a rate of 50 mg L^−1^ N from 15 N–2.2P–12.5 K–2.9Ca–1.2 Mg (JR Peters Inc., Allentown, PA, United States).

Following transplant, 120 ml inoculum of MBSA-MJ1 was applied weekly to each treatment plant. Inoculum was prepared by diluting an overnight culture grown in LB media (OD_595_ = 0.8) 1:100 in reverse osmosis (RO) water as described previously (Nordstedt et al., 2020). Uninoculated LB media diluted in water served as the negative control. Plants were subjected to water stress at 5 weeks post-transplant by temporarily ceasing all irrigation and bacterial treatments until all plants showed a loss of turgidity in their leaves (wilted). To assess recovery, plants were then rewatered with clear water (i.e., no fertilizer) and weekly bacterial treatments were resumed. Plant growth and quality after recovery from water stress were evaluated at 8 weeks post-transplant, which was approximately 2 weeks after the plants were rewatered. These parameters were evaluated by counting flowers (open flowers plus buds showing color) on each plant and collecting shoots (flowers, leaves, and stems) to measure total dry weight. Root-adhering potting media were removed from the roots, and root tissue was collected separately from shoot tissue for dry weights. Root and shoot tissue were dried at 49°C for at least 96 h prior to dry weight measurements.

### *In vitro* Characterization of Strain MBSA-MJ1

#### Growth Under Osmotic Stress

*Serratia plymuthica* MBSA-MJ1 was evaluated for its ability to grow under polyethylene glycol (PEG)-induced osmotic stress conditions according to Nordstedt et al. (2020). Briefly, overnight cultures of MBSA-MJ1 were used to inoculate Yeast Extract Mannitol (YEM) media amended with 30% PEG_8000_. After 96 h incubation, OD_595_ was measured as an indicator of growth under osmotic stress conditions.

#### ACC Deaminase Activity

Enzyme activity of 1-aminocyclopropane-1-carboxcylic acid (ACC) deaminase by *S. plymuthica* MBSA-MJ1 was evaluated using a colorimetric assay according to Nordstedt and Jones (2020). *Pseudomonas putida* UW4 (formerly *Enterobacter cloacae* UW4) was used as the positive control for ACC deaminase activity (Shah et al., 1998), and an uninoculated sample was used for the negative control. Briefly, cultures of MBSA-MJ1 and UW4 were incubated in tryptic soy broth (TSB) for 48 h, then bacterial cells were collected by centrifugation, washed in Dworkin and Foster (DF) media, resuspended in DF media containing ACC as the sole nitrogen source, and incubated for an additional 24 h. After incubation, cells were again collected by centrifugation, triple washed with Tris-HCl, and resuspended in Tris-HCl supplemented with toluene. The samples were added to wells of a microtiter plate containing 0.5 M ACC and incubated in a 30°C water bath for 15 min. HCl was added to the samples and the cells were again collected by centrifugation. The supernatant was mixed with HCl and 2,4-dinitrophenylhydrazine, and the samples were incubated in the water bath for 30 min. NaOH was added to the samples and the absorbance was measured at 540 nm to quantify levels of α-ketobutyrate, the product of ACC degradation by ACC deaminase.

#### Antibiotic Resistance

To test antibiotic resistance of *S. plymuthica* MBSA-MJ1, 0.5X LB agar plates were prepared and supplemented with 1X and 0.5X concentrations of each antibiotic, with 1X concentrations as follows: rifampicin (50 μg/ml), streptomycin (50 μg/ml), kanamycin (50 μg/ml), ampicillin (100 μg/ml), chloramphenicol (25 μg/ml), gentamicin (25 μg/ml), carbenicillin (100 μg/ml), and tetracycline (5 μg/ml). Cells of MBSA-MJ1 from overnight culture were collected by centrifugation, resuspended in phosphate-buffered saline (PBS) buffer, and 5 μl was struck out onto each antibiotic media in triplicate (*n* = 3). Plates were incubated at 28°C for 96 h and then growth on each antibiotic was recorded as yes or no.

#### Motility

*In vitro* motility was evaluated according to Sun et al. (2006). Overnight culture of *S. plymuthica* MBSA-MJ1 was diluted to OD_595_ = 0.8 and then stabbed in nutrient yeast glucose agar (NYGA) semi-solid agar media in triplicate (*n* = 3). The stabbed culture was incubated at 28°C for 96 h, at which point motility was determined by the ability of the culture to grow away from the inoculation stab.

### Whole-Genome Sequencing of Strain MBSA-MJ1

The whole-genome sequence of *S. plymuthica* MBSA-MJ1 was generated to gain insight into the strain’s biology and potential mechanisms to withstand osmotic stress and stimulate the growth of plants during recovery from water stress. Genomic DNA of *S. plymuthica* MBSA-MJ1 was extracted from an overnight liquid culture using the Quick-DNA Bacterial Miniprep kit (Zymo Research, Irvine, CA, United States) and then submitted to Diversigen (formerly CoreBiome; St. Paul, MN) for whole genome shotgun sequencing using the Illumina NovaSeq platform similar to Nordstedt and Jones (2020). Annotation files generated by Prokka (Seemann, 2014) were searched for genes putatively involved in bacterial stress tolerance and plant growth promotion. Kyoto Encyclopedia of Genes and Genomes (KEGG) pathway annotation and mapping was conducted using BlastKOALA (v2.2; Kanehisa et al., 2016). Secreted proteins were predicted using the SignalP webserver (v5.0; Almagro Armenteros et al., 2019), and PlasmidSPAdes was used for detection of plasmid sequences within the genomic data (Antipov et al., 2016).

### Taxonomic Classification and Phylogenetic Comparison

Taxonomy was assigned to *S. plymuthica* MBSA-MJ1 using the Microbial Genomes Atlas (MiGA) because of the program’s ability to compare a query sequence against all taxonomically classified taxa in the NCBI prokaryotic genome database (Rodriguez-R et al., 2018). MiGA assigns taxonomy based on the average nucleotide identity (ANI) concept, which requires >94% ANI to classify two sequences as the same species (Konstantinidis and Tiedje, 2005).

Following taxonomic classification, phylogenetic relationships were established using reference genome sequences of 22 *S. plymuthica*, 13 other *Serratia* spp., and eight closely-related species (according to MiGA ANI results) from the order Enterobacterales. The 13 additional *Serratia* spp. strains were chosen to represent one strain for each *Serratia* species in the NCBI genome database. The ANI values were based on BLAST and calculated using the webtool Enveomics (Rodriguez-R and Konstantinidis, 2016). Distance was estimated using the neighbor-joining clustering method. The Newick output files from the Enveomics ANI program were used to construct the phylogenetic tree using TreeDyn (Chevenet et al., 2006). Additionally, ANI matrix values between the *Serratia* strains were used to determine clades within the phylogenetic tree. Strains that shared the highest level of ANI similarity were grouped, with each clade consisting of strains that each share at least 94% ANI with each other. Strains that do not share at least 94% ANI with any of the strains were not assigned to a clade.

### Statistical Analysis

Statistical analyses for the production-scale greenhouse trial were conducted in R Studio version 3.5.2 using an ANOVA with the model: Y = *μ* + treatment + block. The Tukey’s HSD test was used to determine statistical significance between MBSA-MJ1 treatment and the negative control. Each plant species was analyzed independently of each other.

### Data Availability

The bacterial genome sequence data were deposited with NCBI under BioSample number SAMN16442191.

## Results

### Plant Growth Promotion Under Water Stress

*Serratia plymuthica* MBSA-MJ1 was initially identified as a bacteria that promoted plant growth in *Petunia* × *hybrida* “Picobella Blue” after recovery from water stress in a high throughput greenhouse trial (data not shown). MBSA-MJ1 application increased total shoot dry weight by an average of 14% and increased flower number by an average of three flowers per plant.

**Figure 1:**
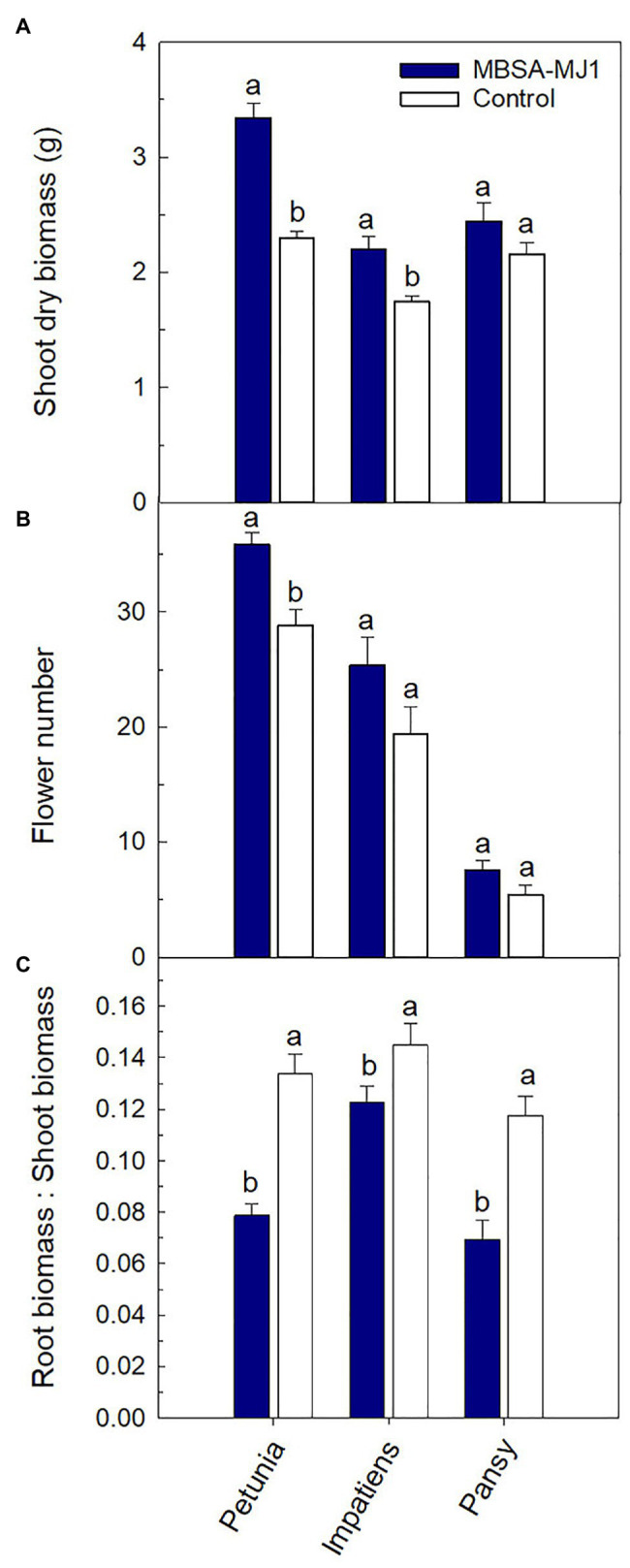
Application of *S. plymuthica* MBSA-MJ1 improved plant growth performance parameters for *Petunia* × *hybrida* (petunia), *Impatiens walleriana* (impatiens), and *Viola* × *wittrockiana* (pansy) plants after recovery from severe water stress. Petunia, impatiens, and pansy plants had 13, 18, and 14 replicates, respectively. Plants were treated weekly with *S. plymuthica* MBSA-MJ1 (blue) or uninoculated LB (control; white). Inoculum was prepared by diluting bacteria culture (OD_595_ = 0.8) or LB media in water 1:100, and 120 ml was applied as a media drench. Plants were grown for 5 weeks and then subjected to severe water stress by withholding all irrigation and bacterial treatment until plants were visibly wilted. Total shoot biomass (dry weight; **A**) and total number of flowers **(B)** were measured 2 weeks after recovery from water stress (8 weeks after transplant). Root:shoot **(C)** was calculated with root and shoot dry weights. Bars represent the mean (± SE), and different letters indicate significant differences between the bacteria treatment and control as determined by the Tukey honest significant difference statistical test.

**Figure 2:**
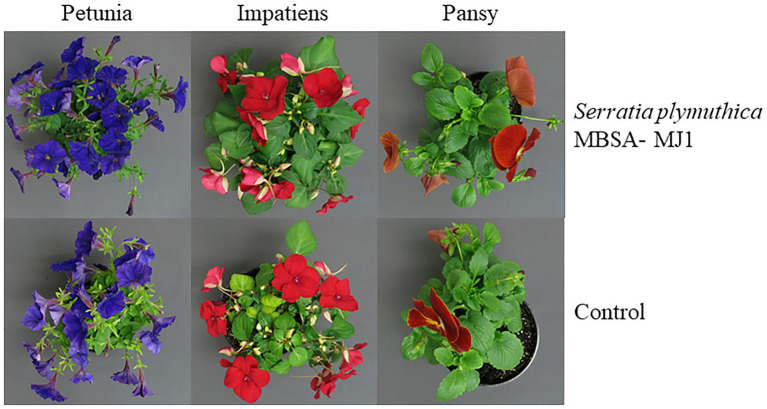
Application of *S. plymuthica* MBSA-MJ1 increased the visual quality of *Petunia* × *hybrida* (petunia) and *I. walleriana* (impatiens) plants, but not *Viola* × *wittrockiana* (pansy) plants, after recovery from water stress. Plants were treated weekly with *S. plymuthica* MBSA-MJ1 or uninoculated LB (control). Inoculum was prepared by diluting bacteria culture (OD_595_ = 0.8) or LB media in water 1:100, and 120 ml was applied as a media drench. Plants were grown for 5 weeks and then subjected to severe water stress by withholding all irrigation and bacterial treatment until plants were visibly wilted (approximately 7 days). Irrigation and weekly treatments were then resumed until week 8 when observations of visual quality were evaluated.

In this manuscript, we present experiments evaluating the growth promoting effects of MBSA-MJ1 on petunia, impatiens, and pansy by assessing growth and flowering after plants were subjected to severe water stress. Application of MBSA-MJ1 significantly increased the shoot biomass of petunia and impatiens compared to the uninoculated control. The greatest effect was observed with petunia, which had an average increase of 45% compared to the uninoculated control plants after recovery from severe water stress. Impatiens and pansy had an average increase in shoot biomass of 26 and 13%, respectively, when treated with MBSA-MJ1, although the difference was not statistically significant in pansy (Figure 1A). Bacterial application also had a beneficial effect on flower number, but this increase was only statistically significant in petunia. Petunia and impatiens treated with MBSA-MJ1 had an average increase of seven and six flowers per plant, respectively, and no difference was observed in pansy (Figure 1B). Petunia, impatiens, and pansy plants treated with MBSA-MJ1 had a 41, 15, and 41% reduction in the root:shoot, respectively (Figure 1C). At approximately 2 weeks after the drought stressed plants were rewatered, petunia and impatiens treated with MBSA-MJ1 were visibly healthier than control plants. Treated plants had darker green foliage, while the foliage of untreated plants was lighter green with more chlorotic leaves (Figure 2). Visual differences between the treated and control pansies were not as obvious.

### *In vitro* Characterization

*Serratia plymuthica* MBSA-MJ1 was able to grow under PEG-induced osmotic stress with an average OD_595_ = 0.45. ACC deaminase activity was not detected in cultures of MBSA-MJ1 (Supplementary Table S1). Antibiotic resistance of MBSA-MJ1 was confirmed by its ability to grow on 1X and 0.5X concentrations of ampicillin, chloramphenicol, and tetracycline, and on 0.5X concentration of carbenicillin. MBSA-MJ1 did not show resistance to rifampicin, streptomycin, kanamycin, or gentamycin. The motile nature of MBSA-MJ1 was demonstrated by its ability to grow away from the inoculation stab in semi-solid agar media.

### Genome Assembly and Annotation

*De novo* assembly and quality filtering of the Illumina reads resulted in 69 contigs with > 1,000 bp length. The draft genome being reported consists of 5,472,087 bases with an average GC content of 56.11%. A total of 5,097 CDSs were identified, with 3,807 (75%) of them being putative protein-coding genes with predicted functions, and 660 identified by SignalP as secreted proteins (Table 1). The KEGG pathway analysis annotated 3,262 genes, 64% of all CDSs, involved in various metabolic pathways. Of the genes assigned to different KEGG functional categories, the largest numbers belonged to the protein families, including genetic information processing (12.7%), signaling and cellular processing (12.1%), and environmental information processing (11.4%). Genes assigned to carbohydrate and amino acid metabolism categories made up 10 and 6.4% of the total gene assignments, respectively (Table 2). No plasmids were identified in the genome sequence data of MBSA-MJ1.

**Table 1:** Assembly and annotation statistics of *Serratia plymuthica* MBSA-MJ1.

Assembly statistics	
Number of bases	5,472,087
Number of contigs (>1,000 bp)	69
N_50_ read length	165,546
GC content	56.11%
Annotation statistics	
Open reading frames (ORFs)	5,206
Number of CDSs	5,097
Protein-coding genes with function prediction	3,807
Protein-coding genes without function prediction	1,290
Secreted proteins	660
Genes assigned to KEGG	3,262

**Table 2:** Kyoto Encyclopedia of Genes and Genomes (KEGG) functional categories of *S. plymuthica* MBSA-MJ1.

Functional category	Annotations	Percent total
Protein families: genetic information processing	415	12.7%
Protein families: signaling and cellular processing	394	12.1%
Environmental information processing	372	11.4%
Carbohydrate metabolism	327	10.0%
Amino acid metabolism	210	6.4%
Unclassified: metabolism	208	6.4%
Unclassified	184	5.6%
Genetic information processing	184	5.6%
Metabolism of cofactors and vitamins	145	4.4%
Cellular processes	112	3.4%
Energy metabolism	106	3.2%
Nucleotide metabolism	101	3.1%
Protein families: metabolism	100	3.1%
Unclassified: signaling and cellular processing	86	2.6%
Lipid metabolism	74	2.3%
Unclassified: genetic information processing	57	1.7%
Glycan biosynthesis and metabolism	52	1.6%
Metabolism of other amino acids	47	1.4%
Human diseases	35	1.1%
Metabolism of terpenoids and polyketides	22	0.7%
Xenobiotics biodegradation and metabolism	22	0.7%
Organismal systems	6	0.2%
Biosynthesis of other secondary metabolites	3	0.1%

### Taxonomic Classification

**Figure 3:**
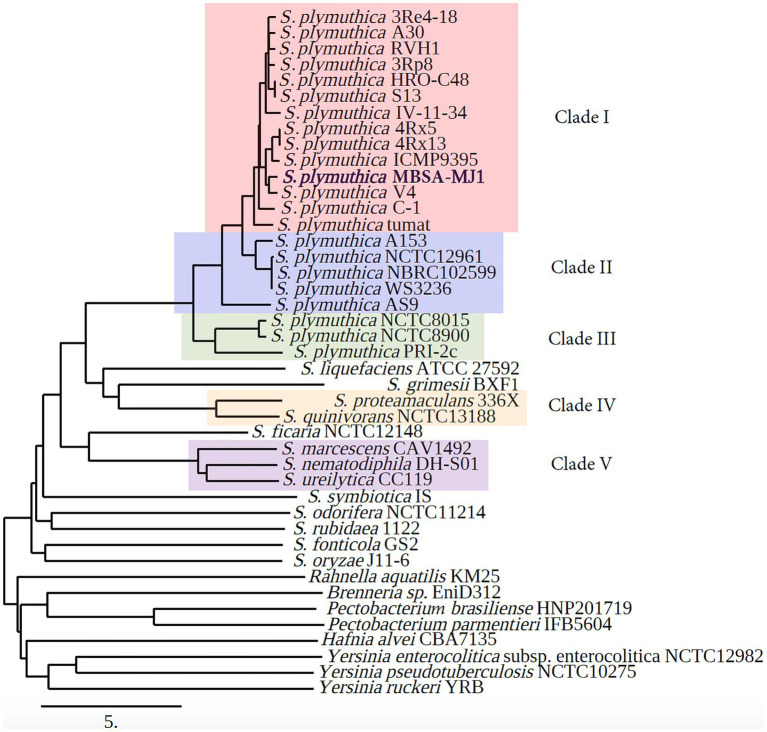
Phylogenetic tree based on average nucleotide identity (ANI) shows that *S. plymuthica* MBSA-MJ1 groups with other *S. plymuthica* strains. The ANI was calculated using whole genome sequences downloaded from NCBI and distance was estimated using neighbor-joining as the clustering method with the Enveomics webtool. The ANI data were also used to establish clades within the different phylogenetic clusters. Strains that shared the highest level of ANI similarity were grouped, with each clade consisting of strains that share at least 94% ANI with each other. Strains that do not share at least 94% ANI with any of the strains were not assigned to a clade. The different clades are indicated as Clade I (red), Clade II (blue), Clade III (green), Clade IV (yellow), and Clade V (purple). The NCBI Biosample number for each genome with the ANI statistics is indicated in Supplementary Tables S2 and S3.

Average nucleotide identity results generated by MiGA classify MBSA-MJ1 as *S. plymuthica* as its sequence shares >94% ANI with 20 different *S. plymuthica* strains in the NCBI prokaryotic genome database (Supplementary Table S2). In addition, using the ANI matrix results generated by Enveomics, we established five clades within the phylogenetic tree based on distinguishable and high-level ANI similarity between strains (Supplementary Table S3). *Serratia plymuthica* MBSA-MJ1 is included in clade I, the largest clade in the analysis, with 13 other *S. plymuthica* strains (Figure 3). Several species were not assigned to clades due to only one strain being included for species outside of *S. plymuthica*, and a lack of ANI similarity between these other species.

### Identification of Genes Putatively Involved in Bacterial Osmotic Stress Tolerance

Multiple genes were identified in the genome of *S. plymuthica* MBSA-MJ1 for the regulation of cellular osmolarity including genes encoding for aquaporin Z (*aqpZ*), glycerol uptake (*glpF*), and the biosynthesis of osmoregulated periplasmic glucans (*mdoGH* and *mdoC*; Delamarche et al., 1999; Lacroix et al., 1999; Lequette et al., 2004; Liu et al., 2015). The genome of MBSA-MJ1 also encodes for the ABC transporter responsible for internalizing osmoprotectants (*proVWX*), and six copies of the osmoprotectant uptake protein (*proP*). Genes comprising the two-component system (TCS) involved in coping with osmotic stress (*envZ*/*ompRC*) and its regulator (*mzrA*), and two cellular envelope stress TCSs (*cpxAR* and *baeSR*) were also identified (Supplementary Table S4; Gerken et al., 2009).

The import and accumulation of K^+^ ions is a common microbial response to osmotic stress (Wood et al., 2001; Wood, 2011). The genome of MBSA-MJ1 encodes for the Kdp potassium uptake system (*kdpABCDE*), which includes the *kdpDE* TCS (Kixmüller and Greie, 2012), the K^+^ uptake proteins (*kup* and *trkH*), and the K^+^ antiporters (*chaA* and *nhaP*; Supplementary Table S4; Radchenko et al., 2006; Resch et al., 2011). Na^+^ extrusion systems also ensure a low sodium content in the cytoplasm while still maintaining osmotic balance during stress (Górecki et al., 2014). Multiple genes involved in sodium transport were identified (*nhaA*, *nhaR*, *nhaK*, *nhaB*, *nhaP*, *cyrA*, and *yrbG*; Supplementary Table S4; Bremer and Krämer, 2019). In addition to the substrate specific K^+^ and Na^+^ transporters, the genome of MBSA-MJ1 also encodes for safety valves for the rapid release of ions and organic solutes in a non-specific manner under sudden osmotic shock (*mscL*, *mscM*, and *mscS*; Supplementary Table S4; Edwards et al., 2012; Bremer and Krämer, 2019).

### Identification of Genes Putatively Involved in Oxidative Defense Response

Microbes must be able to overcome plant reactive oxygen species (ROS) defenses to effectively colonize the rhizosphere or endophytically colonize plant tissue. Multiple genes involved in antioxidant production were identified in the genome of *S. plymuthica* MBSA-MJ1 including: superoxide dismutases (*sodA* and *sodC*), ferric uptake regulator (*fur*), hydrogen peroxide catalases (*katA* and *katG*), hydroperoxide reductase (*ahpD*), thiol peroxidase (*tpx*), peroxiredoxins (*bcp* and *tsaA*), peroxidase (*efeB*), nitric oxide dioxygenase (*hmp*), nitric oxide reductases (*norV* and *norW*), the transcriptional regulator (*norR*), nitric oxide sensor (*nsrR*), and thioredoxins (*trxA*, *trxB*, and *trxC*; Supplementary Table S4; Niederhoffer et al., 1990; Hu et al., 1999; Inaoka et al., 1999; Park et al., 2000; Prieto-Álamo et al., 2000; Zeller and Klug, 2006; Cosgrove et al., 2007; Jangiam et al., 2010; Baptista et al., 2012; Perkins et al., 2015; Zhang et al., 2015; Hong et al., 2019). The genome of MBSA-MJ1 also encodes for multiple genes involved in ROS detoxification systems, including five copies of glutathione S-transferases (*gstB*), glutathione peroxidases (*gpx* and *btuE*), the glutaredoxins operon (*grxABCD*), and two copies of the glutathione ABC transporter (*gsiABCD*; Supplementary Table S4; Tamburro et al., 2004; Smirnova and Oktyabrsky, 2005; Arenas et al., 2010; Sánchez-Riego et al., 2013; Johnson and Hug, 2019). Additionally, several key regulators involved in controlling oxidative stresses, such as the stress response sigma factor (*rpoS*) and its regulator (*rssB*), hydrogen peroxide sensor (*oxyR*), and the organic hydroperoxide resistance proteins (*ohrR* and *ohrB*) were also identified (Supplementary Table S4; Zuber, 2009; Battesti et al., 2011; Flores-Cruz and Allen, 2011; Fontenelle et al., 2011).

In addition to encoding for genes involved in ROS defense protection, the genome of MBSA-MJ1 also encodes for multiple universal stress proteins that confer resistance to oxidative stress (*uspABCEG*), multiple heat shock proteins (*grpE*, *htpX*, *hspQ*, and *hslR*), and chaperone proteins (*dnaJ*, *dnaK*, *groES*/*EL*, *clpB*, *cbpM*, and *cbpA*; Supplementary Table S4; Kornitzer et al., 1991; Squires et al., 1991; Gragerov et al., 1992; Korber et al., 2000; Chae et al., 2004; Shimuta et al., 2004; Nachin et al., 2005; Pepe et al., 2020).

### Identification of Genes Putatively Involved in Rhizosphere Colonization

After overcoming the plant’s ROS defense responses, rhizosphere colonization involves different mechanisms which may include flagellar development, quorum sensing, motility, chemotaxis, and biofilm formation (Cole et al., 2017). Flagellar development requires over 50 genes across multiple operons, of which the genome of *S. plymuthica* MBSA-MJ1 encodes for the entire suite involved in flagellar transcriptional activation, biosynthesis, and construction (*flhCD*, *flgAMN*, *flgBCDEFGHIJKL*, *flhBAE*, *fliAZY*, *fliDST*, *fliE*, *fliFGHIJK*, *fliMNOPQR*, *flgMN*, *flgKL*, *fliC*, *fliDST*, *motAB*, *cheAW*, *tar*, *tsr*, and *aer*; Supplementary Table S4; Chilcott and Hughes, 2000; Macnab, 2003). In addition to flagellar development, three TCSs involved in the regulation of quorum sensing, motility, and chemotaxis (*qseCB*, *envZ*/*ompR*, and *rcsCDB*) were identified (Supplementary Table S4; Sperandio et al., 2002; Francez-Charlot et al., 2003). The genome of MBSA-MJ1 also encodes for multiple copies each of the recombinases/integrases involved in surface colonization (*xerC* and *xerD*), the seven essential lipopolysaccharide transport proteins (*lptABCDEFG*), cellulose biosynthesis (*bcsABZC*), and the *clpP* protein involved in surface adhesion (Supplementary Table S4; O’Toole and Kolter, 1998; Zogaj et al., 2001; Martínez-Granero et al., 2005; Villa et al., 2013; Eida et al., 2020).

### Identification of Genes Putatively Involved in Antibiotic Production and Resistance

Genomic analysis identified the presence of the chloramphenicol acetyltransferase (*cat*) gene, an enzyme that provides resistance to chloramphenicol (Agersø et al., 2019), and six copies of the efflux pump contributing to tetracycline resistance (*stp*) within the genome of *S. plymuthica* MBSA-MJ1 (Supplementary Table S4; Ramón-García et al., 2007), corroborating our *in vitro* findings that MBSA-MJ1 grew on chloramphenicol and tetracycline. In addition to the antibiotics tested in our *in vitro* experiment, the genome of MBSA-MJ1 encodes for genes implicated in resistance to bicyclomycin (*bcr*), fosmidomycin (*fsr*), polymyxin (*arnABCD*), and β-lactam multi-drug resistance (*ampC*, *ampR*, and *ampD*; Supplementary Table S4; Lindberg et al., 1985; Bentley et al., 1993; Fujisaki et al., 1996; Miryala and Ramaiah, 2019). Several multidrug resistance and export proteins were identified within the genome (*emrA*, *emrB*, *emrD*, *emrE*, *emrKY*, *mdfA*, *acrAB*-*tolC*-*acrZ*, *mdtABC*, *mdtJI*, *mdtH*, *mdtK*, *mdtL*, and *mdtN*; Supplementary Table S4; Nishino et al., 2009). The genome of MBSA-MJ1 also encodes for the enzyme responsible for 4-hydroxybenzoate biosynthesis (*ubiC*), an antibiotic against plant pathogenic bacteria (Supplementary Table S4; Siebert et al., 1994; Eida et al., 2020).

### Identification of Genes Putatively Involved in Increasing Plant Water Stress Tolerance

Plant growth promoting rhizobacteria can increase plant water stress tolerance using multiple mechanisms, including the synthesis of osmoprotectants and vitamins and by modulating phytohormone levels (Ngumbi and Kloepper, 2016). Genes encoding for the synthesis of the osmoprotectant proline from glutamate (*proABC*), two proline transporters (*proU* and *proP*), the synthesis of the osmoprotectant glycine betaine from choline (*betAB*), and the glycine betaine transport system (*opuCB*) were identified in the genome of *S. plymuthica* MBSA-MJ1 (Supplementary Table S4; Bhargava, 2006; Hilger et al., 2009; Kohler et al., 2015; Zaprasis et al., 2015; Scholz et al., 2016). Genes were also identified for the synthesis of putrescine (*speA*, *speB*, and *speC*), a polyamine involved in osmotic and oxidative stress (Supplementary Table S4; Kurihara et al., 2005).

The genome of MBSA-MJ1 encodes for genes involved in the synthesis of the vitamins thiamin, pyridoxine, biotin, pantothenate, and folic acid. *De novo* synthesis of thiamin requires 12 genes, including biosynthesis of thiazole (*thiFSGH*, *thiI*, and *dxs*), biosynthesis of pyrimidine (*thiC*), linking thiazole and pyrimidine (*thiE*), and thiamin kinases (*thiD*, *thiM*, *thiL*, and *pdxK*; Begley et al., 1999). There are six genes involved in the biosynthesis of the vitamin pyridoxine (*epd*, *pdxA*, *pdxB*, *pdxJ*, *serC*, *dxs*, and *pdxH*). Additionally, individual operons encoding for the biosynthesis of biotin (*bioCHFADB*), pantothenate (*panBCDE*), and folic acid (*folEBKPCA*) were identified (Supplementary Table S4; Cronan et al., 1982; Guillén-Navarro et al., 2005; Burgess et al., 2009). The gene enocoding for ACC deaminase synthase (*acdS*) was not identified in the genome of MBSA-MJ1.

## Discussion

The gram-negative bacteria *S. plymuthica* is a well-known PGPR that has been isolated from numerous important agricultural crops, including oilseed rape, wheat, potato, pumpkin, maize, and rice (Åström and Gerhardson, 1988; Gyaneshwar et al., 2001; Berg et al., 2002, 2006; Garbeva et al., 2012; Müller et al., 2013; Neupane, 2013). Although the plant growth promoting effects of *S. plymuthica* strains have been primarily attributed to controlling biotic stresses by antagonizing pathogens, our work has shown the utility of this species in stimulating plant growth of containerized horticulture crops after recovery from water stress. Here, we demonstrated the ability of *S. plymuthica* MBSA-MJ1 to increase the shoot size and flower number of multiple plant species after recovery from severe water stress. In addition, we have investigated the genomic characteristics of strain MBSA-MJ1 and connected them to possible biological functions by identifying mechanisms that this novel bacterium might be utilizing to survive, colonize, and stimulate plant growth under water stress conditions.

In general, petunia and impatiens plants treated with MBSA-MJ1 had an increase in shoot growth parameters after recovering from severe water stress when compared to the uninoculated control plants. Both petunia and impatiens were visibly larger with greener leaves (Figure 2), and petunia had significantly more flowers compared to the uninoculated controls (Figure 1). Improvements in these important characteristics will increase overall crop quality and result in plants that are still marketable even after recovery from severe water stress. Each of the three species had a significant reduction in root:shoot biomass, indicating that plants treated with bacteria did not respond to stress with excessive root growth. This is important for containerized production as the energy for plant growth can then be diverted to shoot growth and flower development. Our findings are similar to previous work that showed plants treated with *S. plymuthica* DT8 and *Pseudomonas* spp. significantly increased plant biomass while decreasing the root:shoot of plants recovering from water stress (Nordstedt et al., 2020; Zhang et al., 2020).

The overall treatment effect with MBSA-MJ1 was greatest in petunia, while some positive effects were noted in impatiens and less effect was observed in pansy. Previous work evaluating bacterial application on the same plant species subjected to water stress showed a similar trend in flower number where treatment with *Pseudomonas* spp. increased the flower number in petunia and impatiens, but not pansy (Nordstedt et al., 2020). However, this previous work did show an increase in pansy shoot biomass after recovery from water stress when treated with *Pseudomonas poae* 29G9 or *Pseudomonas fluorescens* 90F12-2. This provides evidence that a single bacterial strain may not be effective for broad application in containerized production systems characterized by the production of many different plant species. Continued work is needed to optimize application parameters such as application timing and rate, and the use of a bacterial consortia with different mechanisms of action to have a broader beneficial effect.

The viability of bacterial cells strongly depends on the water content of their surrounding environment, highlighting a challenge that bacteria face in colonizing plant hosts that are subjected to severe water stress (Kappes and Bremer, 1998; Danhorn and Fuqua, 2007). If bacteria are unable to withstand water stress, they lose the ability to positively influence plant health and stress tolerance during this time. Additionally, Vílchez et al. (2016) suggests a correlation between the level of bacterial desiccation tolerance and the level of water stress tolerance they can confer to plants. The *in vitro* PEG assay provided evidence of MBSA-MJ1s ability to grow under osmotic stress conditions. Comparatively, MBSA-MJ1 showed a higher level of growth under PEG-induced osmotic stress than many other PGPR previously reported to increase plant growth under water stress (Asghar et al., 2015; Nordstedt et al., 2020; Nordstedt and Jones, 2020). In addition, multiple genes were identified within the genome of MBSA-MJ1 responsible for regulating cellular osmolarity that are potentially involved in increasing its ability to grow under osmotic stress.

Two-component systems in bacteria sense and respond to different environmental cues by altering gene expression (Tipton and Rather, 2017). The *envZ*/*ompR* TCS plays a vital role in mediating signal transduction in response to osmotic stress and is involved in the uptake of K^+^ ions (Gerken and Misra, 2010; Yuan et al., 2011). Additionally, the *cpxAR* TCS is essential for rhizospheric fitness and growth by helping the bacteria to survive under harsh environments such as severe water stress (Yan et al., 2020). MBSA-MJ1 also encodes for *mzrA* which not only acts as a regulator of the *envZ*/*ompR* TCS without altering its ability to receive and respond to environmental signals, but also links *envZ*/*ompR* to *cpxAR* to further support cellular envelope stress responses (Gerken et al., 2009).

The production of ROS, nitric oxide, and phytoalexins in the rhizosphere by plants is a common defense mechanism during the early stages of plant-microbe interactions (Hammond-Kosack and Jones, 1996; Zeidler et al., 2004). Therefore, PGPR must be able to withstand this highly oxidative environment in order to colonize the rhizosphere and ultimately stimulate plant growth and stress tolerance. Accordingly, we identified a large variety of genes in the genome of MBSA-MJ1 that encode for antioxidants, ROS detoxification, stress response factors that play a key role in controlling oxidative stresses, and universal stress proteins that confer resistance to oxidative stress.

Survival in the rhizosphere also depends on the bacterium’s ability to outcompete other organisms. A common mechanism for this is the production or resistance to antibiotics and other antimicrobial compounds (Andrés-Barrao et al., 2017). Our *in vitro* work demonstrated MBSA-MJ1’s resistance to different antibiotics, and our genomic analyses identified genes involved in resistance to antibiotics not tested in our *in vitro* work that could be further contributing to MBSA-MJ1s fitness in the rhizosphere. In addition, the production of secreted proteins has been attributed to many *Serratia spp*. ability to survive in diverse environmental conditions (Petersen and Tisa, 2013), and a large number of genes encoding for secreted proteins were also identified within the genome of MBSA-MJ1.

The ability to move in a controlled manner confers distinct advantages to rhizospheric bacteria, particularly during colonization. Flagellar development plays a vital role in the motility of bacteria especially during times of environmental stress, and it has been established that nonmotile bacteria are severely impaired in colonizing and surviving within the rhizosphere (Lugtenberg et al., 2001; Capdevila et al., 2004; Guo et al., 2020). However, the bacteria flagellar motor is the most complex structure of the bacterial cell, requiring over 50 genes across multiple operons, of which the MBSA-MJ1 genome contains the entire suite of genes (Chilcott and Hughes, 2000; Armitage and Berry, 2020). These findings corroborate the results from the *in vitro* motility experiment that confirmed MBSA-MJ1’s ability to move spatially in semi-solid agar media. Chemotaxis is another important trait in competitive colonization as it controls the direction of flagellar rotation and movement (De Weert et al., 2002; Danhorn and Fuqua, 2007; Guo et al., 2020), and the three chemotaxis-related TCS identified in the genome of MBSA-MJ1 likely play a fundamental role in this complex function. PGPR that colonize the rhizosphere have also been known to colonize plant tissue as endophytes (Santoyo et al., 2016). Considering that multiple *S. plymuthica* strains have been previously identified as endophytes (Benhamou et al., 2000; Liu et al., 2011; Müller et al., 2013), coupled with our genetic evidence in MBSA-MJ1 for flagellar development and chemotaxis, and the positive results from the *in vitro* motility assay, it is probable that MBSA-MJ1 can also colonize plant tissue as an endophyte. However, this is yet to be experimentally proven.

This work has also identified a multitude of putative mechanisms that this PGPR might be utilizing to stimulate plant growth and increase stress tolerance. Similar to bacteria, plants respond to osmotic stress by synthesizing or accumulating compatible solutes, such as trehalose, mannitol, proline, and glycine-betaine, which increase plant stress tolerance and the ability to grow under water stress conditions (Bhargava, 2006). The *proABC* operon is responsible for synthesizing the osmoprotectant proline, and the proline transporters *putP* and *proY* could provide a mechanism for bacterial-synthesized proline to be made available to the plant host (Bhargava, 2006; Hilger et al., 2009; Kohler et al., 2015; Zaprasis et al., 2015). Additionally, MBSA-MJ1 has the potential to synthesize the osmoprotectant glycine-betaine, which is encoded by *betAB*, and export the compound into the rhizosphere *via* the *betT* high-affinity choline transport system (Scholz et al., 2016). The compatible solute carnitine has been shown to improve plant recovery during times of salt stress and has been implicated in modulating the ABA pathway in plants (Charrier et al., 2012). The six copies of the *proP* carnitine transport system encoded in the genome of MBSA-MJ1 indicate that carnitine might be another compatible solute involved in increasing the water stress tolerance of plants. Further, the presence of genes involved in synthesizing the polyamine putrescine (*speABCE*) adds to the list of osmoprotectants that might be involved in this beneficial plant microbe interaction (Kurihara et al., 2005).

Bacterial-produced vitamins also play an important role in plant growth and development and stress responses. Therefore, it is beneficial for plants to form symbiotic relationships with rhizospheric bacteria that can synthesize vitamins for them (Palacios et al., 2014). Our genomic analyses identified multiple genes within the genome of MBSA-MJ1 involved in the synthesis of different B-vitamins, a class of bacterial-produced vitamins that has been attributed to plant growth promotion and increased stress tolerance (Deryło and Skorupska, 1993). Thiamine (vitamin B1) functions as an important enzyme cofactor in carbohydrate and amino acid metabolism and has previously been shown to increase the growth of clover (Deryło and Skorupska, 1993; Schyns et al., 2005). Riboflavin (vitamin B2) is required for the production of cofactors involved in cellular metabolism and is required for normal plant growth and development (Bereswill et al., 1999; Deng et al., 2014). In particular, riboflavin has been shown to enhance plant growth by increasing production of photosynthetic pigments, increasing carbon assimilation, and even increasing tolerance to osmotic stress (Palacios et al., 2014). Riboflavin has also been shown to increase water stress tolerance and antioxidant enzyme activity of tobacco (Deng et al., 2014). Pyridoxine (vitamin B6) is required by all living organisms and acts as a potent antioxidant that effectively quenches ROS (Bilski et al., 2000; Palacios et al., 2014). Pyridoxine has been shown to play a role in plant oxidative stress response (Mooney et al., 2009) and has demonstrated potential function in regulating Na^+^ homeostasis and thus conferring salt-stress tolerance to plants (Shi et al., 2002). Niacin (vitamin B3) is involved in nearly every metabolic pathway in the cell, with its most important function being as cofactor for diverse cellular oxidation–reduction reactions under stress conditions that might lead to increased stress tolerance in plants (Noctor et al., 2006; Palacios et al., 2014). Biotin and pantothenic acid are also involved in carrying out essential metabolic functions in plants, although they have not yet been evaluated for their influence on plant stress tolerance (White et al., 2001; Guillén-Navarro et al., 2005).

Overall, this work has identified *S. plymuthica* MBSA-MJ1 as an effective plant growth promoter of containerized horticulture crops subjected to severe water stress. Although *Serratia* spp. have been extensively studied for control of biotic stresses, there is still much work to be done to understand how they increase abiotic stress tolerance in plants. The comprehensive genome analyses conducted in this study have identified potential genes in the genome of MBSA-MJ1 whose protein products may function in pathways involved in MBSA-MJ1’s ability to survive, colonize, and eventually stimulate plant growth under severe water stress. Future work should focus on functional characterization of the genes identified here in order to begin elucidating the mechanisms most prominently involved in the beneficial responses observed in our greenhouse trials. In addition, the formulation of consortia treatments with other bacterial strains identified as PGPR should be considered as a viable solution to achieving broad beneficial effects across different plant species.

## Data Availability Statement

The datasets presented in this study can be found in online repositories. The names of the repository/repositories and accession number(s) can be found at: www.ncbi.nlm.nih.gov/, PRJNA669647.

## Author Contributions

NN and MJ conceived the experimental design. NN performed the greenhouse production trial, *in vitro* assays including collecting and analyzing the data, and all genomic analyses, and led the writing of the manuscript. MJ acquired funding and resources, served as project administrator, and edited the manuscript. Both the authors contributed to the article and approved the submitted version.

### Conflict of Interest

The authors declare that the research was conducted in the absence of any commercial or financial relationships that could be construed as a potential conflict of interest.
